# Complete Active Space Methods for NISQ Devices: The Importance of Canonical Orbital Optimization for Accuracy and Noise Resilience

**DOI:** 10.1021/acs.jctc.3c00123

**Published:** 2023-04-27

**Authors:** Juan Angel de Gracia Triviño, Mickael G. Delcey, Göran Wendin

**Affiliations:** † Department of Microtechnology and Nanoscience - MC2, Chalmers University of Technology, SE-412 96 Gothenburg, Sweden; ‡ Division of Theoretical Chemistry and Biology, Department of Chemistry, Royal Institute of Technology, SE-114 28 Stockholm, Sweden; § Division of Theoretical Chemistry, Department of Chemistry, Lund University, SE-223 62 Lund, Sweden

## Abstract

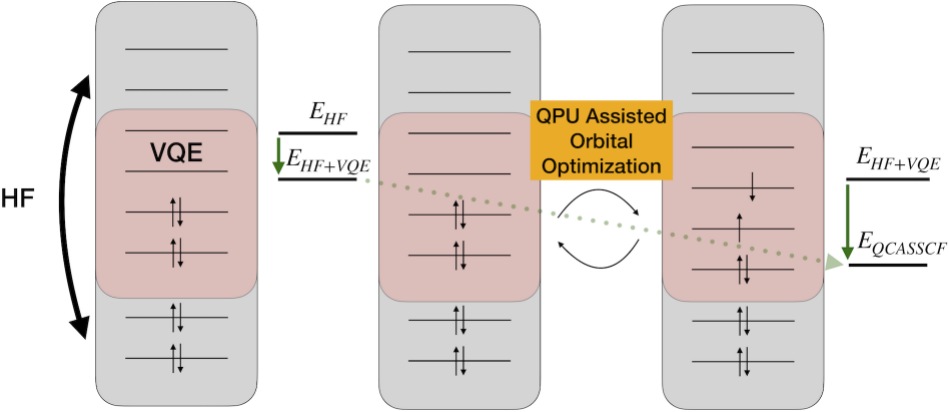

To avoid the scaling of the number of qubits with the size of the basis set, one can divide the molecular space into active and inactive regions, which is also known as complete active space methods. However, selecting the active space alone is not enough to accurately describe quantum mechanical effects such as correlation. This study emphasizes the importance of optimizing the active space orbitals to describe correlation and improve the basis-dependent Hartree–Fock energies. We will explore classical and quantum computation methods for orbital optimization and compare the chemically inspired ansatz, UCCSD, with the classical full CI approach for describing the active space in both weakly and strongly correlated molecules. Finally, we will investigate the practical implementation of a quantum CASSCF, where hardware-efficient circuits must be used and noise can interfere with accuracy and convergence. Additionally, we will examine the impact of using canonical and noncanonical active orbitals on the convergence of the quantum CASSCF routine in the presence of noise.

## 1 Introduction

Theoretical chemistry has always been one of the main drivers of the development of high-performance computing and computers (HPC)^1,2^ and is now aiming at saturating the resources of exascale computers.^3,4^ Since the calculation of electronic structure and dynamics is ultimately NP-hard, this means that classical computing may soon face an exponential wall, not the least when it comes to energy consumption, information is physical.^5^ Here, quantum computers may in principle provide exponential advantage for quantum chemistry through quantum superposition and entanglement. The original “killer application” was Shor’s algorithm for factorization^6^ but is now rather defined by computation of energy level structures of catalyzing enzymes like FeMoCo with chemical accuracy.^7^ Exponential computational advantage is however an elusive concept that might be difficult to achieve in practice.^8^ In the NISQ era, we therefore need to settle for more modest goals, programming quantum chemistry applications on quantum computers and trying to achieve practical quantum advantage.

Quantum computational chemistry algorithms can be roughly divided into two categories: quantum phase estimation (QPE)^9^ and hybrid variational quantum algorithms (VQA). QPE has two important limitations: the first one is the need to have an initial state with nonzero overlap with the true FCI eigenstate, which requires a state preparation routine to increase the probability of collapsing in the ground state.^10,11^ The second limitation comes from the need for an increasing number of ancilla register qubits (in addition to the Hamiltonian qubits) to reach the desired accuracy.^11,12^ The iterative QPE^13,14^ requires only one register qubit, but its implementation will be limited by the coherence time in the quantum device.^13^ On the other hand, VQAs and, particularly, the variational quantum eigensolver (VQE)^15^ reduce significantly the requirements for coherent times in the quantum device.^16^ The VQE is based on the Rayleigh-Ritz variational principle:
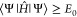
1where Ψ is the molecular electronic wave function,  is the molecular Hamiltonian operator, and *E*_0_ is the ground state energy. From here, the VQE will use a parametric version of the molecular electronic wave function  where  denotes a vector of parameters. Consequently, the problem is reduced to finding the parameters that minimize the energy. The next natural step will be bringing the molecular Hamiltonian into an adequate form to be used in the quantum device. To that end, an especially convenient form of the Fermionic Hamiltonian is the second quantized Hamiltonian:
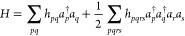
2where the one-body integral:
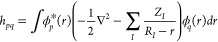
3and the two-body integral:
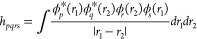
4are expressed on molecular orbital (MO) basis ϕ(*r*), and *a*^†^, *a* are the creation and annihilation operators, respectively. The MOs can be obtained from either the Hartree–Fock (HF) method or from density functional theory (DFT). Eq 2 can be mapped to a qubit Hamiltonian using encoding methods like Jordan–Wigner (JW)^17^ or Bravyi–Kitaev (BK)^18^ after transformation of the integrals (eq 3 and 4) from MO basis to spin–orbital (SO) basis. An SO can contain only one electron, and the occupation number can be stored in the qubit states |0⟩ for unoccupied or |1⟩ for occupied. After the mapping, the electronic Hamiltonian will be expressed as a linear combination of products of single-qubit Pauli operators:
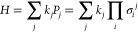
5where *k*_*j*_ are real scalars, σ is a Pauli operator *X*, *Y*, *Z* or the identity operator *I*, and *P* are known as Pauli strings and represent the product of single-qubit operators. At this point, the length of the Pauli strings, which correspond to the number of qubits of the Hamiltonian, is dependent on the number of SOs, which is also dependent on the atomic basis set size by the following relations:

- Number of contracted atomic basis functions = Number of MOs
- Number of SOs = 2 × Number of MOs
- Number of qubits = Number of SOs*

*This relation only holds if no qubit reduction by symmetries is used during the mapping.

To illustrate this dependence, we can take water as an example: As can be seen in Table 1, the size of the basis set increases the number of qubits in the Hamiltonian. In consequence, the required number of qubits in the quantum device for larger molecules with large basis sets increases dramatically and becomes intractable in the current generation of noisy intermediate-scale quantum (NISQ) devices.^19^ Consequently, proposals for quantum computational chemistry have been focused on isolating the strongly correlated component of the molecule to be treated by the quantum device, known in chemistry as active space.^20^ In this study, we will focus on several of the strategies proposed in the literature to increase the accuracy without additional hardware requirements. We will specifically investigate three strategies: First, just constraining the number of qubits by using an active space for the qubit Hamiltonian (eq 5) and describing classically how the choice of the basis set affects the ground state energy (Section 2). The second strategy will be to relax (optimize) the active space through a classical complete active space self-consistent field (CASSCF) calculation^21^ to see how the basis set affects the ground state energy (Section 2). Finally, the third approach will be a hybrid quantum CASSCF similar to the one proposed by Tilly et al.^22^ where the orbital optimization will be done by dividing tasks between classical and quantum backends (Section 3). This last strategy, being the most noise-sensitive (relying on accurate densities to converge), will be explored in both noiseless and noisy simulators (Section 4).

**Table 1:** Number of Qubits Dependence with the Basis Set Size for Water

Basis set	Contracted basis functions	Qubits
STO-3G	7	14
6-31G	13	26
CC-PVDZ	24	48

## 2 Nonorbital-Relaxed Active Space: How Is the Accuracy Related to Enlarging the Basis Set?

**Figure 1:**
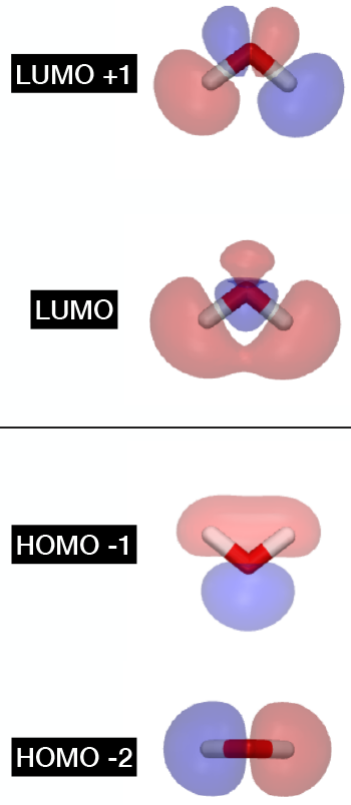
Orbitals considered in the active space for water

As mentioned in the Introduction, increasing the basis set size leads to longer Pauli strings in the qubit Hamiltonian. The choice of an active space is a straightforward way to reduce the qubit requirements in the quantum backend and allow the use of larger basis sets without additional qubits. Naively, one might expect an increase in the accuracy with a larger basis set, but it is unclear how the result is distributed between the classical and the quantum calculations. To reveal the accuracy dependence on the basis set, we will consider the water molecule (geometry in Supporting Information) and the following basis sets: STO-3G,^23^ 6-31G,^24^ CC-PVDZ^25^ and ANO-L-VDZP.^26,27^ We will restrict the active space to 4 MOs leading to 8 qubits (Figure 1: HOMO–2, HOMO–1, LUMO, and LUMO+1; the HOMO is excluded: it corresponds to the oxygen atom lone pair and does not have significant correlation with any other valence orbital). The integrals and molecular orbitals will be computed through an HF calculation using the quantum chemistry package VeloxChem^28^ and MultiPsi (article in preparation). Once the integrals are obtained, the molecular Hamiltonian will be mapped to a qubit Hamiltonian using the JW encoding, and the minimum eigenvalue is computed using VQE with a unitary coupled cluster singles-doubles (UCCSD) ansatz^29^ with an HF initial state; all those included in the package IBMQ Qiskit.^30^ The UCCSD-VQE minimum eigenvalue will account for the active space energy, and the energy of the inactive space is described by HF. The total UCCSD-VQE energy will be the addition of active and inactive space energies. In this work we will employ the Löwdin definition of correlation,^31^ i.e., difference with respect to HF.

The results in Table 2 show how, as expected, the HF energy does improve with increasing basis set. It is important to bear in mind that, besides CC-PVDZ and ANO-L-VDZP having the same number of contracted basis functions, the better result in the latter comes from the number of primitive basis functions (34 in CC-PVDZ versus 101 in ANO-L-VDZP) and the use of general contraction. On the other hand, the correlation obtained by UCCSD-VQE energy decreases, with the energy almost identical to the HF energy for the largest basis. The same trend can be observed when using a classical CASCI for the active space. The decrease in the correlation energy as the basis set becomes larger indicates that the increased accuracy is due to the classical HF calculation and not the quantum UCCSD-VQE – the situation is dominated by the classical HF result. This is mostly due to the poor shape of the HF virtual orbitals in large basis sets: the canonical orbitals tend to converge to something that is more suitable to describe the electron attachment (typically more diffuse, details in Supporting Information) but less suitable to describe correlation. Consequently, using a nonrelaxed-orbital (nonoptimized) reduced active space (to be computed with the quantum backend) and increasing the quality of the basis set provokes the confluence of the UCCSD solution with the HF solution for a fixed active space. Evidently, increasing the active space will increase the energy difference between UCCSD and HF to include more correlation, but the trend is expected to stay the same since the relative ratio of inactive/active space will increase with the basis set size, increasing the HF contribution to the final energy.

**Table 2:** Ground State Energy for Water in Different Basis Sets Computed Both by a Classical HF and via UCCSD-VQE^a^

Basis set	Number basis	qubits	HF Energy (Ha)	UCCSD-VQE Energy (Ha)	Correlation (Ha)	CASCI (Ha)
STO-3G	7	8	–74.960337	–74.991216	0.030878	–74.991249
6-31G	13	8	–75.983339	–75.986901	0.006333	–75.989779
CC-PVDZ	24	8	–76.026984	–76.028769	0.002941	–76.029973
ANO-L-VDZP	24	8	–76.054374	–76.055219	0.001112	–76.055493

a In addition, correlation and the classical CASCI solution are included.

### 2.1 Effect of Using an Orbital-Relaxed Wave Function for the Quantum Backend Simulation

To increase the correlation in the active space and improve the accuracy of the quantum simulation, one can bring more correlated orbitals into the molecular Hamiltonian. This strategy has been adopted before by Sugisaki et al.^21^ to study the insertion of beryllium into H_2_ but, in our case, we are interested in the trend of the correlation with the basis set size. We have used the same molecule with the same active space as before, but, in this case, we have obtained the integrals from a converged CASSCF calculation computed with MultiPsi.

In Table 3, the energies obtained by using a converged CASSCF wave function are compared to the energies obtained by using a HF wave function. In general, orbital optimization is required to correctly describe the static correlation in the active space, especially important for larger basis sets than the minimal STO-3G. This is due, again, to the poor shape of the virtual orbitals for describing correlation and orbital optimization will change the shape of the orbitals to maximize the correlation energy (see Supporting Information). In consequence, an orbital optimization routine is required for any meaningful calculation using an active space to reduce the qubit requirements.

**Table 3:** Comparison between the HF Energy and the UCCSD-VQE Energy Obtained Using Nonrelaxed Orbitals and Relaxed Orbitals^a^

Basis set	Number basis	Qubits	HF Energy (Ha)	Nonrelaxed orbitals (Ha)	Relaxed orbitals (Ha)	Correlation increase (Ha)
STO-3G	7	8	–74.960337	–74.991216	–75.004076	0.012860
6-31G	13	8	–75.983339	–75.986901	–76.035091	0.048190
CC-PVDZ	24	8	–76.026984	–76.028769	–76.076806	0.048037
ANO-L-VDZP	24	8	–76.054374	–76.055219	–76.103328	0.048109

a The last column contains the increase in the correlation energy due to the orbital optimization.

Classically optimizing the orbitals, while being promising, does not exclude the need to perform a CASSCF calculation, in which case the converged energy will also be slightly better when using a full CI (FCI) description of the active space instead of UCCSD (Table 4). The difference between the classical CASSCF and the UCCSD will be more significant the bigger the active space is and it will be zero for the CAS(2,2) case since UCCSD is exact in the case of 2 molecular orbitals for including single and double excitations. In order to avoid performing a classical CASSCF calculation that would render the UCCSD-VQE irrelevant, we will explore a quantum CASSCF routine that better utilizes the quantum device capabilities.

**Table 4:** Ground State Energy for Water in Different Basis Sets Computed Both by a Classical HF and via UCCSD-VQE Using a CASSCF(4,4) Wave Function

Basis set	Number basis	Qubits	HF Energy (Ha)	UCCSD-VQE Energy (Ha)	Correlation (Ha)	Exact CASSCF (Ha)
STO-3G	7	8	–74.960337	–75.004076	0.043739	–75.004111
6-31G	13	8	–75.983339	–76.035091	0.051753	–76.035113
CC-PVDZ	24	8	–76.026984	–76.076806	0.049822	–76.076824
ANO-L-VDZP	24	8	–76.054374	–76.103328	0.048954	–76.103337

## 3 Quantum CASSCF Routine

**Figure 2:**
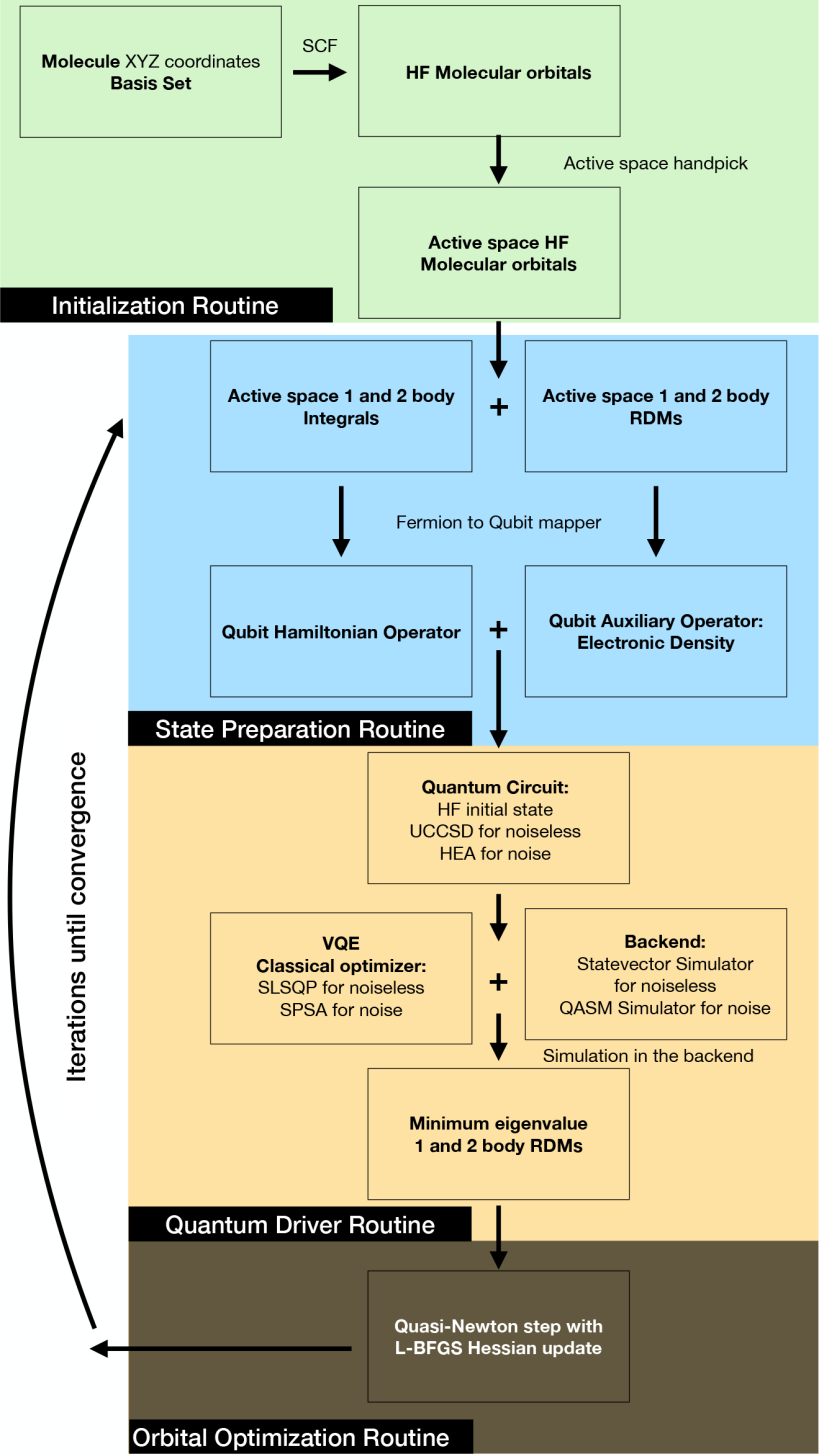
Flowchart of the overall quantum CASSCF routine. The algorithm is divided into the initialization routine to generate the initial guess (green). The state preparation routine (blue) that maps the integrals and RDMs to qubit operators. The quantum driver routine (orange) builds the circuit and compute the minimum eigenvalue and RDMs using VQE. The orbital optimization routine (brown) will use the results from the quantum driver to optimize the orbitals and the new integrals and RDMs will come back to the state preparation routine and continue the cycle until convergence.

The idea of a hybrid orbital optimization routine has been explored first by Takeshita et al.,^20^ and a fully hybrid quantum-classical CASSCF has been reported by Tilly et al.^22^ where the 1 and 2 body RDMs were sampled independently to mitigate their error and noncanonical orbitals were used. Other quantum multi-configurational SCF implementations have been also reported in the literature.^29,32−37^ Nevertheless, to our knowledge, no comparison has been made with the results coming from an orbital-optimized wave function in the quantum measurement after VQE. We have implemented a CASSCF routine (Figure 2) that evaluates the 1 and 2 body RDMs as auxiliary operators to the Hamiltonian and uses canonical CASSCF orbitals (and thus in particular natural orbitals within the active space) by default.

### 3.1 Numerical Simulations on Water

The intent of simulating the quantum CASSCF in a noiseless statevector simulator is to explore the potential of this method in terms of accuracy. Therefore, we will use the same chemically inspired ansatz (UCCSD with HF initial state) and the 4 orbitals 4 electrons active space used until now. In this case, only the ANO-L-VDZP basis set will be used in order to benchmark the energy obtained with other references. Also, tight convergence criteria (Δ*E* < 1*e*^–8^ and ) have been settled for the numerical simulations. To accelerate the convergence, we can restart the calculation from another converged calculation using a smaller active space. In consequence, we have used a converged CASSCF(2,2) obtained with the hybrid routine to restart the CASSCF(4,4). Our reference energies for benchmarking will be the reported ones for water considering the full space in STO-6G basis set (−75.728533 Ha) where also 8 qubits were used and in 6-31G (−76.118828 Ha) where 20 qubits were used.^19^ For completeness, the convergence will be compared with a classical CASSCF where a FCI driver is used in the active space.

**Figure 3:**
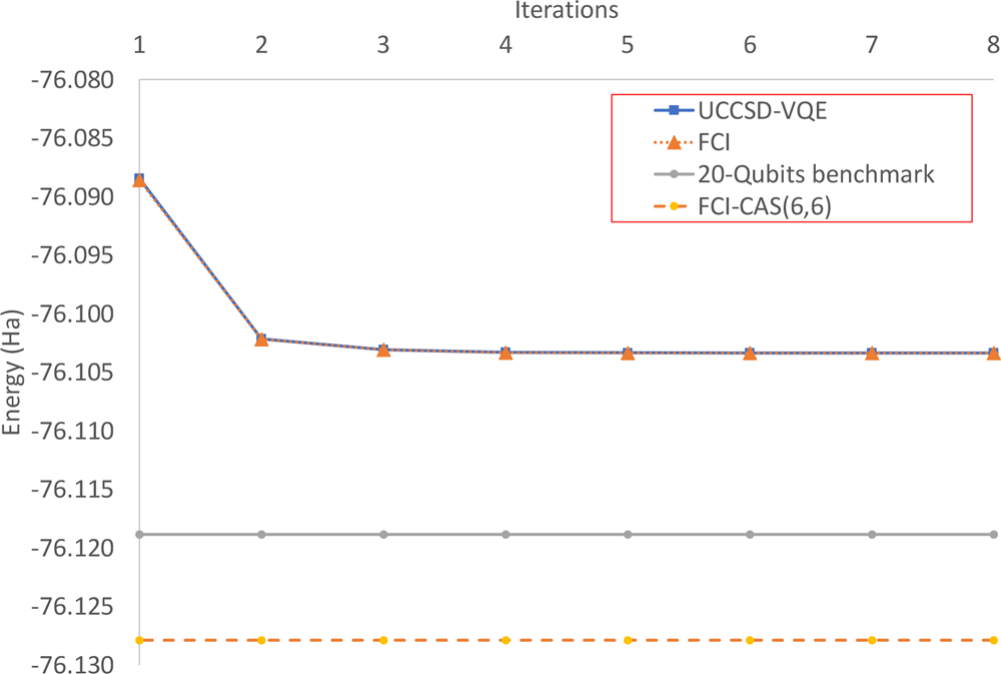
Convergence CASSCF(4,4) with the UCCSD-VQE driver and a classical FCI driver. For comparison, it also shows the 20 qubits reported energy^19^ as benchmark and the value for a CASSCF(6,6) on the ANO-L-VDZP basis set.

In Figure 3 we can see that the UCCSD-VQE driver and the FCI driver converge in the same fashion and to almost the same energy (7.8*e*^–6^ Ha higher than FCI). The final converged energy is −76.103329 Ha, which is only 0.015499 Ha higher than the 20 qubits reported.^19^ In order to obtain a lower energy than the 20 qubits reported, one could use this method with a larger CAS(6,6) and the expected energy would be slightly higher than or equal to −76.127864 Ha. The lack of significant difference between both drivers is expected to be due to the weak correlation of the water molecule, and FCI does not offer a significant advantage with respect to UCCSD. This hypothesis will be properly tested in the next section. Also, the final energy is just 1.3*e*^–6^ Ha lower than the one obtained by using the orbital-optimized wave function directly in the quantum backend. This means that in accuracy terms, there is no significant gain in using a quantum CASSCF routine. Also, using a converged CASSCF wave function in the quantum backend is expected to be less sensitive to quantum noise since it relies on a single measurement. In addition, by applying the reference state error-mitigation proposed by Rahm and collaborators,^38^ the energy could be easily corrected. Nevertheless, classical CASSCF algorithms present a factorial scaling of computational resources with respect to the active space size.^22^ Therefore, while this strategy is feasible for small active spaces, for larger active spaces a quantum CASSCF could potentially overcome this scaling limitation.^39^

### 3.2 Numerical Simulations on Strong Correlation: Cyclobutadiene Automerization Transition State

**Figure 4:**
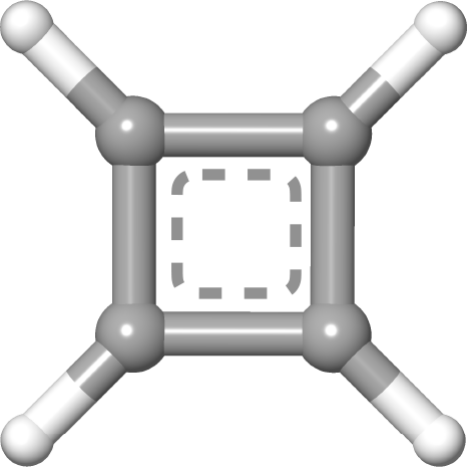
Representation of the transition state of the butadiene automerization.

The transition state in the cyclobutadiene automerization (geometry in Supporting Information) is known for having a strong correlated transition state defined by a open-shell *D*_4*h*_ symmetry, as seen in Figure 4. We will compute the transition state energy using the same settings as used for water, and the selected orbitals will be HOMO–1, HOMO, LUMO and LUMO+1 (π and π*) corresponding to a CAS(4,4).

**Figure 5:**
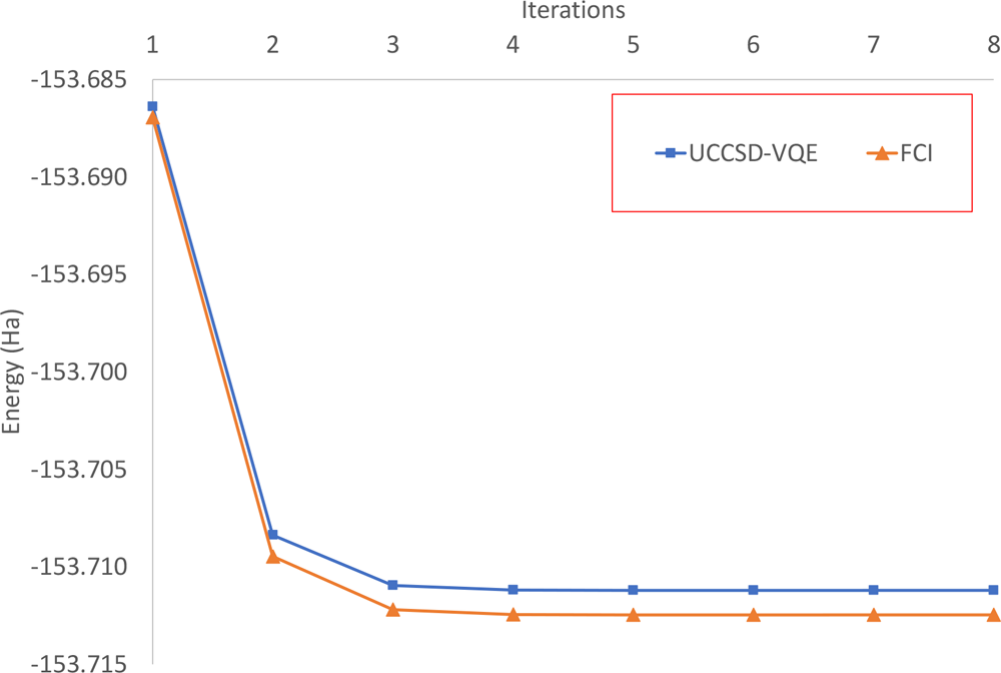
Convergence CASSCF(4,4) with the UCCSD-VQE driver and a classical FCI driver for the cyclobutadiene automerization transition state.

As shown in Figure 5, the UCCSD-VQE driver does not reach the FCI driver energy. While in water for a CAS (4,4) active space the UCSSD energy differs with the FCI energy 7.8*e*^–6^ Ha, for cyclobutadiene (with the same active space size), the error increases to 1.2*e*^–3^ Ha. This loss of accuracy is due to the stronger correlation in the cyclobutadiene active space that will need higher-order excitations than doubles to describe its correlation exactly. Nevertheless, the energy difference 1.2*e*^–3^ Ha is in the chemical accuracy (1.6*e*^–3^ Ha) range, and consequently this method is still highly accurate.

### 3.3 Theoretical Performance in Other Strongly Correlated Molecules

Even though the error with respect to FCI is larger for the transition state of the cyclobutadiene automerization than for water, the error is still better than chemical accuracy. Since for strongly correlated molecules the accuracy is more sensitive to the inclusion of higher order excitations, other strongly correlated molecules will be tested with the quantum CASSCF routine and compared to classical CASSCF. In particular we will test three systems with increasingly bigger active space: CO (stretched to 1.54 Å, singlet CAS(2,2)),^22^ HO_3_ (doublet CAS(3,3)) and C_2_ (singlet CAS(4,4)) using the CC-PVDZ basis set. All the geometries can be found in the Supporting Information. As shown in Table 5, with bigger active space, the error increases. For the CAS(2,2) and CAS(3,3) systems, the error is in the same order of magnitude. For the CAS(4,4), the error increases by 2 orders of magnitude. We additionally performed a quantum CASSCF on C_2_ with a CAS(6,5) active space, and we can see how the error increase another order of magnitude. Even though C_2_ is a strongly correlated molecule (the occupation numbers for the CAS(4,4) active space are 1.917, 1.001, 0.999 and 0.083) similar to the cyclobutadiene automerization transition state (the occupation numbers for the CAS(4,4) active space are 1.904, 1.000, 1.000 and 0.096) for the same active space, the error is lower. The reason is the correlation in C_2_ has relatively strict pair structure, well reproduced by UCCSD, while the one in cyclobutadiene does not, which can be seen when looking at the conventional CI coefficients (see Supporting Information).

**Table 5:** Comparison between the Converged Energies of CO, HO_3_ and C_2_ Obtained by Our Implementation of a Quantum CASSCF (UCCSD-VQE-CASSCF) and the Exact CASSCF Using a FCI Description of the Active Space^a^

Molecule	Active Space	Qubits	UCCSD-VQE-CASSCF (Ha)	Exact CASSCF (Ha)	Error (Ha)
CO (1.54 Å)	CAS(2,2)	4	–112.587151284	–112.587151556	2.72*e*^–7^
HO_3_	CAS(3,3)	6	–224.359615960	–224.359616324	3.64*e*^–7^
C_2_	CAS(4,4)	8	–75.472752753	–75.472778601	2.58*e*^–5^
C_2_	CAS(6,5)	10	–75.516391471	–75.516662232	2.72*e*^–4^

a The error in Hartrees is indicated in the last column. As an exception, this table presents the energies with 9 decimal positions instead of 6 to represent the difference between both methods. An additional row with a CAS(6,5) active space for C_2_ is added for representing the increase in the error with an additional molecular orbital.

## 4 Quantum CASSCF in Noisy Simulations

**Figure 6:**
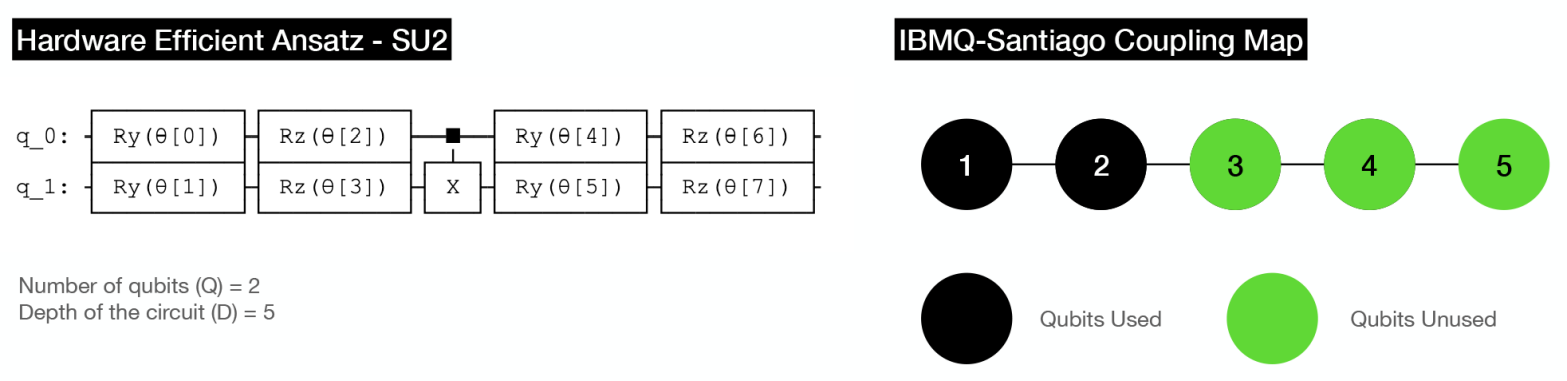
On the left side the hardware efficient ansatz used in the noisy simulations and on the right side the coupling map of IBMQ-Santiago corresponding to the *Canary r3* processor type and a quantum volume of 32.^44^

So far, all the results have been produced under the assumption of ideal behavior of the quantum device. In contrast, NISQ devices are noisy, which creates decoherence and computational errors. We have tested the performance of the quantum CASSCF under noisy simulations, including realistic noise coming from IBMQ Santiago version 1.3.14 (details in the Supporting Information). In this way, we tested convergence with 1000 shots in the IBMQ QASM Simulator using the coupling map from IBMQ Santiago (Figure 5, right). The choice of the IBMQ Santiago device was motivated by ensuring testing our implementation under the same backend conditions as the implementation by Tilly et al.^22^ It can be assumed unrealistic to use tight convergence criteria in the presence of noise, and for these simulations, very loose convergence criteria have been established (Δ*E* < 1*e*^–5^ and ). In the same way, the UCCSD ansatz has been replaced with a shallow HEA represented in Figure 6 (left) to avoid amplifying the noise due to the circuit depth, but at the cost of less theoretical accuracy (−76.063804 Ha for HEA^40^ vs −76.068513 Ha for UCCSD in numerical simulations). The optimizer for the VQE parameters has been replaced from the sequential least squares programming (SLSQP) to simultaneous perturbation stochastic approximation (SPSA)^41^ for having a better performance in the presence of noise.^42^ Finally, we have also used a parity mapper with two-qubit reduction.^43^ To test the convergence under noisy simulations for water, we have considered a smaller active space consisting of the HOMO–1 and the LUMO (2 electrons and 2 orbitals).

### 4.1 Reduced Density Matrices and Canonical Orbitals

The 1-body reduced density matrix 1-RDM  is defined as
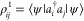
6and the 2-body reduced density matrix (2-RDM)  as
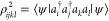
7Focusing on the 1-RDM, some properties are of interest:^45^
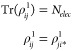
8where *N*_*elec*_ is the number of electrons. Similarly, normalization and symmetries^45^ can be imposed in the 2-RDM:
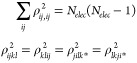
9These properties have been imposed for mitigating errors due to noise in the 1-RDM and 2-RDM.

Additionally, diagonalization of the 1-RDM leads to canonical MCSCF orbitals (also known as natural orbitals) and are used by default in MultiPsi and Dalton,^46^ whereas this is not the default in the CASSCF implementation of PySCF.^47^ The previous report from Tilly and co-workers^22^ does not indicate the use of canonical orbitals, and diagonalizing the 1-RDM is an additional transformation (to be added to normalization and symmetry) for which we do not know if it mitigates or amplifies errors. The 1-RDM transformations routine for canonical orbitals will undergo the following steps after readout from the quantum backend and prior to the orbital optimization:

- 1-RDM evaluated in the quantum simulator
- Normalization of the 1-RDM as
- Symmetrization as
- Canonicalization as , where *U* is the unitary matrix of eigenvectors
- Orbital optimization routine

The 1-RDM transformations routine for noncanonical will just skip the canonicalization step and use  directly in the orbital optimization routine.

### 4.2 Convergence with Canonical vs Noncanonical Orbitals

In order to investigate the effect of canonicalization in the quantum CASSCF routine, we will test the convergence with canonical and noncanonical orbitals. In noiseless numerical simulations, the choice of canonical or noncanonical orbitals does not change the final converged energy (−76.063804 Ha). Since the noise will induce random behavior in the energies and density matrices, the quantum CASSCF has been executed 20 times for each type of orbitals in the active space with the aim to extract averaged tendencies.

**Figure 7:**
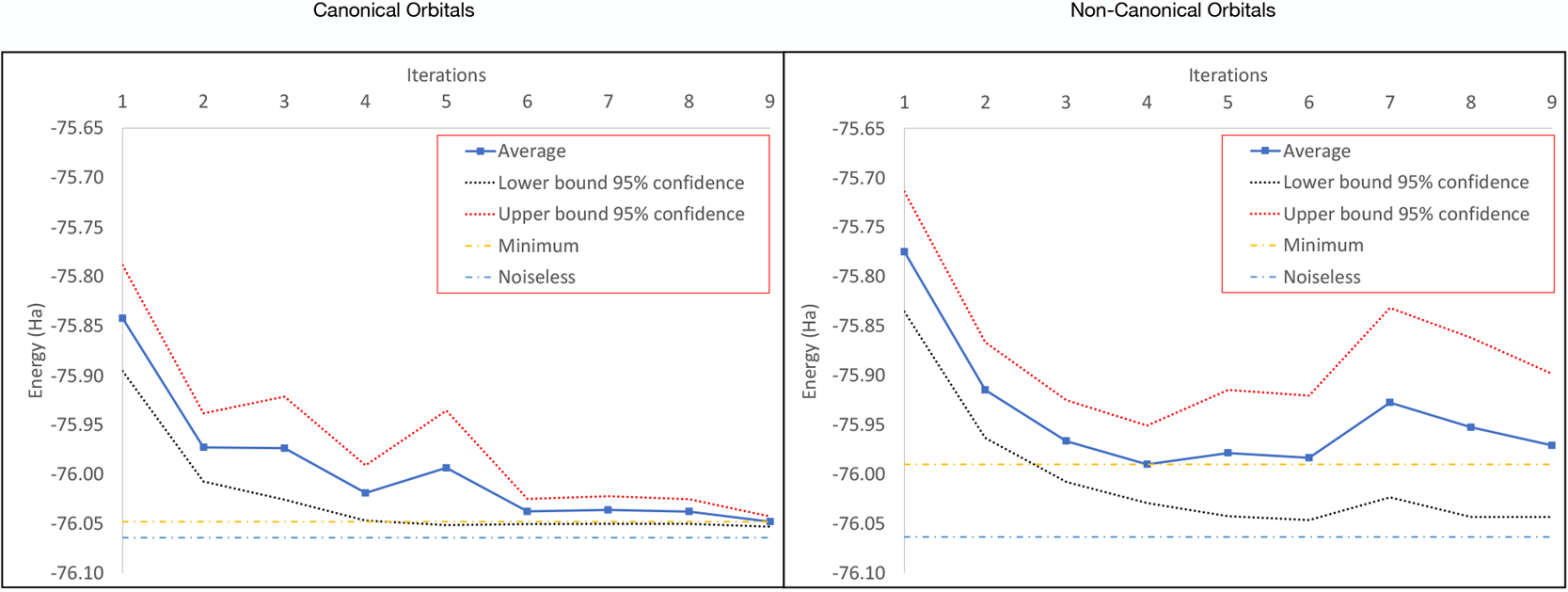
On the left side is shown the averaged convergence for 9 iterations of the quantum CASSCF(2,2) for water with the HEA-VQE driver using canonical orbitals and on the right side is shown the averaged convergence with noncanonical orbitals. In both cases, the 95% confidence interval has been added and compared the minimum average energy with the with the noiseless converged energy.

From Figure 7 it is clear that the use of canonical orbitals significantly reduces the effect of noise on the convergence of the quantum CASSCF without any further error mitigation technique. Moreover, toward the last steps, the deviation decreases significantly (indicating convergence), whereas, with noncanonical orbitals, the opposite occurs – the deviation increases. It is also noticeable that for the case of noncanonical orbitals, the minimum energy is achieved around the fourth iteration, and the following iterations tend to have larger deviations indicating deconvergence. In both cases, the number of iterations were truncated to 9 since for canonical orbitals, the convergence was reached after a few steps (different for every repetition) and for noncanonical orbitals, an oscillating behavior was observed. Therefore, 9 was the maximum number of iterations that allowed a statistically well represented comparison between both types of orbitals.

As shown in Table 6, not only the convergence is poorer with noncanonical orbitals but also the minimum energies produced are worse. This result, even though it does not constitute any error mitigation procedure, reveals that some error mitigation can be achieved on the orbital optimization side, at least for this particular molecule.

**Table 6:** Minimum Average Energies, Averaged Standard Deviation for All the Data Points and Averaged 95% Confidence over All the Data Points for Water^a^

Method	Min. Energy (Ha)	Avg. Std. Deviation	Avg. Confidence 95% (Ha)
Canonical	–76.047720	0.068468	0.030007
Noncanonical	–75.990149	0.146013	0.063992
Noiseless	–76.063804	–	–
FCI	–76.068513	–	–

a Included also the FCI exact energy for the CASSCF(2,2).

### 4.3 Convergence for a Strongly Correlated Molecule

**Figure 8:**
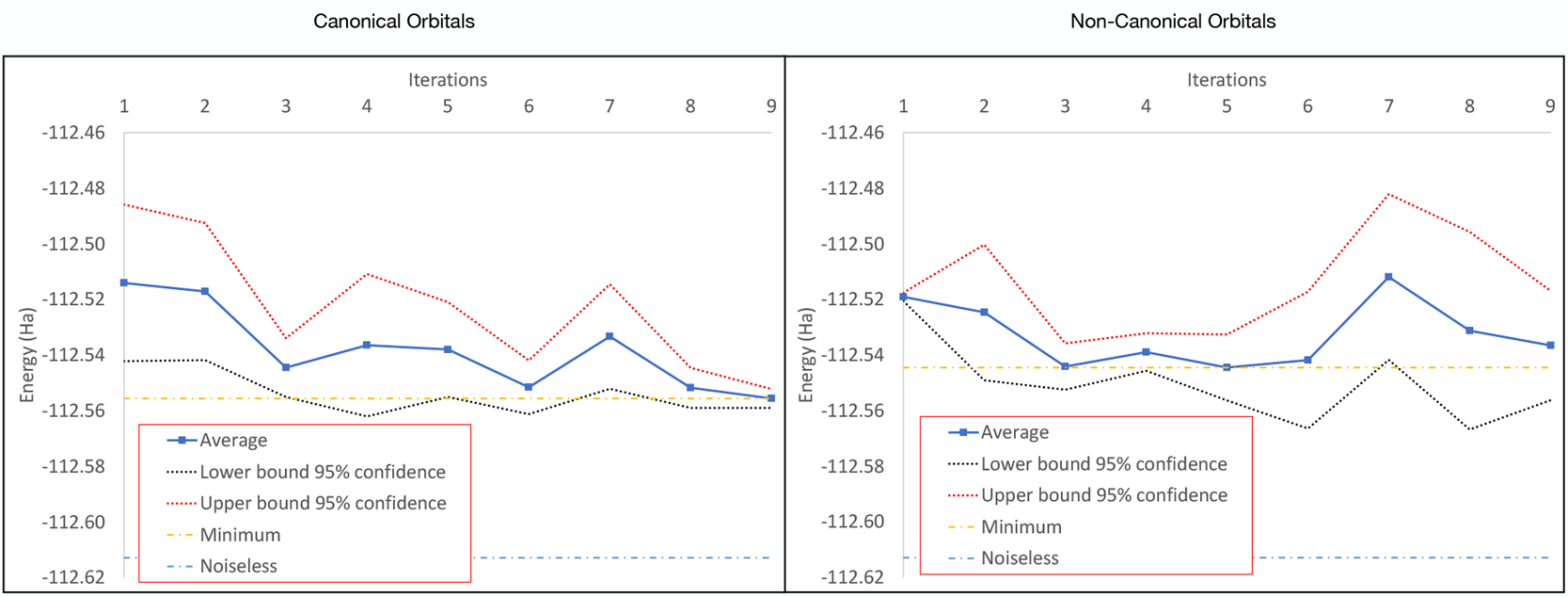
On the left side is shown the averaged convergence for 9 iterations of the quantum CASSCF(2,2) for CO with the HEA-VQE driver using canonical orbitals and on the right side is shown the averaged convergence with noncanonical orbitals. In both cases, the 95% confidence interval has been added and compared the minimum average energy with the with the noiseless converged energy.

Using the quantum CASSCF routine (Section 4), strongly correlated molecules have also been tested. A larger error of the UCCSD-VQE with respect to the FCI solver compared to water was then observed. To elaborate on this, we performed an additional comparison between canonical and noncanonical orbitals for a strongly correlated CO molecule. In particular, we studied CO stretched to 1.54 Å in CC-PVDZ basis in order to have a CAS(2,2) active space. In Figure 8 it can be observed that the convergence patterns between canonical and noncanonical are less different than in the case of water. Nevertheless, they reflect the same trend as observed for water, namely, convergence for canonical orbitals and deconvergence for noncanonical. Also, like in the case of water, the minimum value for noncanonical orbitals is achieved earlier (5th iteration), and from that iteration the deviation increases. From Table 7 can be seen quantitatively that the difference between canonical and noncanonical is not as acute as observed for water. This is reflected in the difference in average minimum energy (around 0.01 Ha) and the dispersion of the data measured as the average standard deviation and average 95% confidence. Regardless of these similarities, the results show a decrease in the dispersion of the data in the last iterations and a more marked convergence profile for canonical orbitals. The accuracy of the noisy simulations is not as close to the noiseless value (−112.587151 Ha) as in the case of water, but, on the other hand, the HEA ansatz presents the same accuracy as the UCCSD ansatz (−112.587151 Ha) for this particular case in the numerical simulation. The reason for this may be the stronger correlation (HOMO occupation is 1.747 and LUMO occupation is 0.253 computed with both UCCSD and HEA) in such a small active space is well described by the simplified entanglement in the HEA ansatz (represented by a CX gate). For water, the occupation numbers obtained by the UCCSD driver are 1.988 for HOMO and 0.012 for the LUMO, while with the HEA driver are 1.992 for HOMO and 0.008 for LUMO (the FCI occupations are 1.988 for HOMO and 0.012 for LUMO). Therefore, it seems that for the particular case of the stretched CO, the HEA describes well the correlation, while for water UCCSD offers a better description. To get a more general performance benchmark, further studies must be conducted. All these results indicate that the convergence of a quantum CASSCF is complex and depends on the quantum circuit, the correlation of the molecule, the active space size and the choice of orbitals.

**Table 7:** Minimum Average Energies, Averaged Standard Deviation for All the Data Points and Averaged 95% Confidence over All the Data Points for CO^a^

Method	Min. Energy (Ha)	Avg. Std. Deviation	Avg. Confidence 95% (Ha)
Canonical	–112.554696	0.026031	0.016134
Noncanonical	–112.544475	0.029129	0.018054
Noiseless	–112.587151	–	–
FCI	–112.587151	–	–

a Included also the FCI exact energy for the CASSCF(2,2).

## 5 Conclusion

In this work, we explore strategies to improve the accuracy of quantum computational chemistry simulations while keeping the number of qubits low. Specifically, three strategies have been explored, from the simplest of just reducing the active space of the molecule to the most complex relaxing the active orbital space through a quantum CASSCF. The choice of just selecting the active space as the most strongly correlated orbitals without relaxation (optimization) will lead to an eminently classical HF description of the molecule unless the active space is large enough. On the other hand, classically relaxing the orbitals improves significantly the results, but at the cost of performing an actual CASSCF calculation on the classical side. If the CASSCF is to be done with the aid of quantum computers, as in the quantum CASSCF routine presented here, the convergence is strongly affected by noise. Moreover, canonicalization of the active space orbitals appears to greatly improve the convergence in absence of an independent sampling of the RDMs, like the one reported by Tilly and co-workers.^22^ By using canonical orbitals (which is computationally unexpensive), convergence is reached in noisy simulations without error mitigation or sampling of the RDMs.^22^ This indicates that improvements in the orbital optimization routines can be done to mitigate errors without additional cost on the quantum devices. We believe that this work can serve as a starting point to design more noise-tolerant orbital optimization routines.

## Supporting Information Available

Molecular geometries used in this work and details on the composition of the molecular orbitals (PDF)
