# Supplementary material for: Complete Active Space Methods for NISQ Devices: The Importance of Canonical Orbital Optimization for Accuracy and Noise Resilience
Source: J Chem Theory Comput. 2023 Apr 27;19(10):2863–72. doi: 10.1021/acs.jctc.3c00123 (PMC10210242; doi:10.1021/acs.jctc.3c00123)
Supplement: Supplementary file 1 — ct3c00123_si_001.pdf [file ct3c00123_si_001.pdf]

Supporting Information

Complete active space methods for NISQ devices: The importance of canonical orbital optimization for accuracy and noise resilience

Juan Angel de Gracia Triviño[*,†], Mickael G. Delcey[‡,§], and Göran Wendin[†]

[†]*Department of Microtechnology and Nanoscience - MC2, Chalmers University of Technology, SE-412 96, Gothenburg, Sweden*
[‡]*Division of Theoretical Chemistry and Biology, Department of Chemistry, Royal Institute of Technology, SE-114 28, Stockholm, Sweden*
[§]*Physical and Theoretical Chemistry, Department of Chemistry, Lund University, SE-223 62, Lund, Sweden*

E-mail: juan.degracia@chalmers.se

Contents

S1 Molecular geometries **S1**

S2 Molecular orbitals referred in Section 2.2 **S2**

S3 CI coefficients of C_2 and cyclobutadiene referred in Section 3.3 **S3**

S3.1 C_2 . . . . S3

S3.2 Cyclobutadiene S3

S4 IBMQ Santiago additional information **S4**

S5 References **S5**

S1 Molecular geometries

The molecules studied in this article have the following xyz coordinates in Å:

Water:

```
O 0.0000 0.0000 0.0000
H 0.0000 0.7408 0.5821
H 0.0000 -0.7408 0.5821
```

Cyclobutadiene transition state:

```
C -0.7236 -0.7236 0.0000
C 0.7236 -0.7236 0.0000
C -0.7236 0.7236 0.0000
C 0.7236 0.7236 0.0000
H -1.4858 -1.4858 0.0000
H 1.4858 -1.4858 0.0000
H -1.4858 1.4858 0.0000
H 1.4858 1.4858 0.0000
```

CO stretched:

```
C 0.0000 0.0000 0.0000
O 0.0000 0.0000 1.5400
```

HO_3:

```
O -1.2463 -0.1947 -0.3212
H -0.4388 -0.7369 -0.3246
O -1.2201 0.1730 1.1931
O -0.2440 -0.3347 1.7845
```

C_2:

```
C -0.6596 0.0000 0.0000
C 0.6596 0.0000 0.0000
```

Note that the atomic units (Å) have been retained.

S2 Molecular orbitals referred in Section 2.2

The specific molecular orbitals considered for water will be a linear combination of atomic orbitals (LCAO) from the basis set. In order to demonstrate the diffusiveness or unrelaxed molecular orbitals coming from a larger basis set, we will show, for example, the Hartree-Fock LUMO in the STO-3G[1] and ANO-L-VDZP[2,3] basis sets:

STO-3G

(1 O 2s : 0.91) (1 O 1p0 : 0.75) (2 H 1s : -0.81)
(3 H 1s : -0.81)

ANO-L-VDZP

(1 O 2s : -0.90) (1 O 3s : -0.43) (1 O 1p0 : -0.29)
(2 H 1s : 0.68) (2 H 2s : 0.55) (3 H 1s : 0.68)
(3 H 2s : 0.55)

We can observe that in the ANO-L-VDZP basis, the LUMO contains the 3s orbital in the oxygen, lowering the contribution from the p orbital. Also, the hydrogen atoms contain 2s orbitals. If we optimize the orbitals in both basis sets, we can observe how close the Hartree-Fock orbitals are to the optimized ones:

STO-3G

(1 O 2s : -0.90) (1 O 1p0 : -0.76) (2 H 1s : 0.80)
(3 H 1s : 0.80)

The composition of the LUMO in STO-3G has changed very slightly, indicating that the HF guess was not far from the optimized orbitals.

ANO-L-VDZP

(1 O 2s : 0.92) (1 O 1p0 : 0.73) (1 O 2p0 : -0.33)
(2 H 1s : -0.75) (3 H 1s : -0.75)

After optimization, the composition of the LUMO has changed significantly, and the oxygen is composed mainly of 2s and p orbitals (no more 3s contribution), while the hydrogen atoms are again composed solely of 1s orbitals. This result indicates that the relaxation of the molecular orbitals has a stronger effect on the larger basis set, whereas for minimal basis sets, optimization will not significantly improve the ground state energy.

S3 CI coefficients of C_2 and cyclobutadiene referred in Section 3.3

The CAS(4,4) CI coefficients coming from the converged solution of the classical CASSCF are in the following subsections. The reader may notice how in the case of C_2 the determinants shows a localized paired correlation in the double bond whereas for cyclobutadiene the correlation is more disperse and shared between orbitals. This observation is related with the better performance of the UCCSD in C_2 compared to cyclobutadiene.

S3.1 C_2

Table S1: CI coefficients for C_2

Determinant	coef.	weight
2200	-0.692	0.479
2020	0.692	0.479
0202	0.144	0.021
0022	-0.144	0.021

S3.2 Cyclobutadiene

Table S2: CI coefficients for cyclobutadiene

Determinant	coef.	weight
2200	-0.677	0.458
aabb	0.110	0.012
2020	0.677	0.458
abab	-0.110	0.012
baab	-0.221	0.049

S4 IBMQ Santiago additional information

Here we present additional information on the IBMQ Santiago quantum backend. The IBMQ QASM simulator was used, which has been fed with noise and the coupling map from the IBMQ Santiago device. The data corresponds to version 1.3.14, which is publicly available at: https://github.com/Qiskit/qiskit-terra/tree/main/qiskit/providers/fake_provider/backends/santiago

The relevant data for the noise characterization of the two qubits used (qubit 1 and qubit 2) is shown below:

Table S3: Noise characterization of Qubit 1

**Parameter**	**Value**	**Unit**
Frequency	4.83	GHz
Read-out error	0.013	–
Rx-gate error	0.00020	–
Rz-gate error	0	–

Table S4: Noise characterization of Qubit 2

**Parameter**	**Value**	**Unit**
Frequency	4.62	GHz
Read-out error	0.014	–
Rx-gate error	0.00016	–
Rz-gate error	0	–

Additionally, the error associated with the **two-qubit gate Cx (2,1)** is 0.0063. All the data has been added to the IBMQ QASM Simulator to account for the device noise.

S5 References

1. Hehre, W. J.; Radom, L.; Schleyer, P. V. R.; Pople, J. A. *Ab Initio Molecular Orbital Theory*; John Wiley & Sons, Inc.: 1986.

2. Woon, D. E.; Dunning, T. H. Gaussian Basis Sets for Use in Correlated Molecular Calculations. III. The Atoms Aluminum through Argon. *J. Chem. Phys.* **1993**, 98 (2), 1358–1371.

3. Page, C. S.; Olivucci, M.; Merchán, M. A Theoretical Study of the Low-Lying States of the Anionic and Protonated Ionic Forms of Urocanic Acid. *J. Phys. Chem. A* **2000**, 104 (38), 8796–8805.
